# Cost-Effectiveness Analysis of Sintilimab Combined with Chemotherapy Versus Chemotherapy Alone as the First-Line Treatment for Advanced Esophageal Cancer

**DOI:** 10.3389/fphar.2022.934275

**Published:** 2022-11-28

**Authors:** Zhuo-Miao Ye, Zhe Xu, Fan-Yuan Zeng, Zi-Qing Tang, Qin Zhou

**Affiliations:** ^1^ Department of Oncology, Xiangya Hospital, Central South University, Changsha, China; ^2^ National Clinical Research Center for Geriatric Disorders, Xiangya Hospital, Central South University, Changsha, China; ^3^ Department of Pharmacy, Xiangya Hospital, Central South University, Changsha, China

**Keywords:** cost-effectiveness, sintilimab, esophageal cancer, Markov model, PD-L1

## Abstract

**Background:** Esophageal cancer has a poor prognosis and currently ranks sixth in global cancer mortality rates. The ORIENT-15 trial showed sintilimab plus chemotherapy significantly improved survival when compared to chemotherapy alone. This study aimed to evaluate the cost-effectiveness of sintilimab, a programmed death-ligand 1 (PD-L1) inhibitor, plus chemotherapy in treating patients with esophageal cancer compared with chemotherapy alone.

**Methods:** A Markov model with a 10-year horizon was developed based on the perspective of the Chinese healthcare payers. We conducted a cost-effectiveness analysis for sintilimab combined with chemotherapy based on a questionnaire. Patients were grouped into the sintilimab group based on a positive score of 10 or more (combined positive score (CPS) ≥ 10 groups), and those with any other PD-L1 expression were randomized into patient groups. We estimated the cost and the effectiveness of sintilimab on the quality-adjusted life-years (QALYs), and the incremental cost-effectiveness ratio (ICER) was computed. One-way and probabilistic sensitivity analyses were conducted to explore the impact of uncertainties on the cost-effectiveness results.

**Results:** In the base-case analysis, compared with chemotherapy alone, the ICER of sintilimab plus chemotherapy for all patients was $21024.05 per QALY, and in the CPS≥10 group, it was $20974.23 per QALY. This was lower than $37653 per QALY. One-way sensitivity analysis demonstrated that ICERs were most sensitive to the price of sintilimab.

**Conclusion:** The study demonstrated that sintilimab plus chemotherapy for advanced esophageal cancer as its first-line treatment would be more cost-effective than chemotherapy alone in Chinese patients.

## Introduction

Esophageal carcinoma is a prevalent and fatal malignancy consisting of two major histological types: adenocarcinoma and squamous cell carcinoma (SCC). The latter accounts for approximately 90 % of all cases of esophageal carcinoma (Rustgi and El-Serag, 2014; Lagergren et al., 2017). Esophageal carcinoma is widely distributed globally, whereas SCC predominates in Asia, Africa, and South America, and adenocarcinoma is prevalent in North America and Europe (Abnet et al., 2018; Uhlenhopp et al., 2020). In addition, major risk factors include gastroesophageal reflux disease (GERD), cigarette smoking, and consumption of alcohol (Uhlenhopp et al., 2020). The increasing availability of endoscopy screening to identify precancerous conditions and effective patient education has reduced the incidence of esophageal carcinoma in many regions. In the United States, the decline in the incidence rate of esophageal cancer was 1.5 % annually from 2007 to 2016 (Ilson and van Hillegersberg, 2018). However, the prognosis of patients with esophageal carcinoma remains poor, and they are not diagnosed until the disease has reached advanced stage I. Recent studies have revealed that the five-year survival rate ranges 20 − 35%, even if there is no metastasis (Napier et al., 2014). In addition, the current primary treatment for the disease includes surgical intervention, platinum-based chemotherapy, and radiotherapy regimens which have only brought a modest benefit to the overall survival (OS) (Kelly, 2019; Fatehi Hassanabad et al., 2020). As a result, the aggressive nature of esophageal cancer with its early spread, rapid tumor recurrence, and poor prognosis underlines the significance and necessity for innovative medical therapies.

In recent years, immune checkpoint inhibitors targeting programmed cell death protein 1 (PD-1) and programmed cell death ligand 1 (PD-L1) have attracted global attention as a novel therapy in treating numerous malignancies. They are able to effectively reduce regulatory T-cell apoptosis and block the immune escape mechanism of tumors (Iwai et al., 2002; Sun et al., 2018). Among them, sintilimab, a fully recombinant human immunoglobulin G4 (IgG4) anti-PD-1 monoclonal antibody, has been approved as treatment for non-small-cell lung cancer, classical Hodgkin’s lymphoma, and hepatocellular cancer by the National Medical Products Administration of China (Shi et al., 2019; Yang et al., 2020; Ren et al., 2021; Zhou et al., 2021). Sintilimab and several other PD-1 monoclonal antibodies have been proven to act as single-agent activity in patients with esophageal squamous cell carcinoma and given as their first-line chemotherapy, leading to an overall improvement of their outcome (Kato et al., 2019; Huang et al., 2020; Kojima et al., 2020; Xu et al., 2020). ORIENT-15, a phase III clinical trial, demonstrated that the combination of sintilimab with chemotherapy outperformed ongoing first-line treatment (cisplatin plus 5-fluorouracil chemotherapy) in patient survival (Lu et al., 2022). In the clinical trial, the sintilimab plus chemotherapy group displayed better progression-free survival (PFS) with a hazard ratio of 0.56 (95 % CI:0.46 – 0.68) than the control group (Lu et al., 2022). Despite the encouraging clinical performance, the high treatment cost of sintilimab has been under the spotlight. Current cost-effective analyses of sintilimab are mostly conducted for hepatocellular carcinoma and non-small-cell lung carcinoma (Peng et al., 2022; Zhou et al., 2022; Zhu et al., 2022). Studies on the cost-effectiveness of sintilimab combined with chemotherapy as the first-line treatment for esophageal carcinoma need to be conducted. Therefore, this study aimed to investigate the cost-effectiveness of sintilimab as the primary treatment for patients diagnosed with locally advanced, recurrent, or metastatic esophageal squamous cell carcinoma from the perspective of China health-care payers.

## Materials and Methods

### Population

The target cohort for this study was based on the patient characteristics from the population studied in the phase III ORIENT-15 clinical trial. The factors included patients ≥ 18 years and a pathological diagnosis of locally advanced or metastatic esophageal squamous cell carcinoma. A total of 676 subjects were included in this study. The experimental group (327 patients) received sintilimab plus chemotherapy combination therapy and the control group (332 patients) received chemotherapy plus placebo. There were no statistical differences in the baseline characteristics of the patients in the sintilimab plus chemotherapy group and the chemotherapy group. The data used in this study were obtained from public data. Therefore, patient consent and study approval from an Ethics Committee and Institutional Review Committee were not required.

### Markov Model Structure

The study used TreeAge software 2021 (TreeAge Software, Inc., Williamstown, Massachusetts) to program a multi-state Markov model to evaluate the cost-effectiveness of sintilimab plus chemotherapy compared with chemotherapy. The multiple health states include PFS, progressive disease state (PD), and death (Supplementary Figure S1). If patients in a certain state only make one state transition in a cycle, then patients in the PD state cannot return to the PFS state. Similarly, if patients have died then they cannot transition to other states (Ding et al., 2021). The specific transition relationships are shown in Supplementary Figure S1. We assumed that all patients included in this study were in a healthy PFS state at the initial stage of the model. Patients were randomized into two groups: sintilimab with or without chemotherapy. When the disease progressed, the follow-up treatment plan in the ORIENT-15 clinical trial was used as an additional treatment until the patient’s death.

The Markov cohort was used to simulate the patients’ entire live courses. With reference to the dosing cycle from the ORIENT-15 clinical trial, we set the cycle of the Markov model to three weeks, and the time horizon of the model was set at 10 years. Approximately 99 % of the patients died after model simulation. A half-circle correction was conducted to simulate the transfer process more accurately. Simultaneous simulation analysis of the cost and utility of the therapy was performed to compute the cumulative total cost and health utilities within the cohort’s time frame (She et al., 2019). The research was based on the Chinese health-care payers’ perspective with a 3 % discount on costs and utilities of the treatment (Sanders et al., 2016). According to the World Health Organization, the incremental cost-effectiveness ratio (ICER) was acceptable when it is below three times the gross domestic product (GDP) per capita. This study will use three times of China’s GDP per capita in 2021 (US $37653) as the threshold. The willingness-to-pay (WTP) was assumed to be $37653. The research indicators included the cost, life-years (LYs), quality-adjusted life-years (QALYs), and ICERs. The research methods conformed and referred to the consolidated health economic evaluation reporting standards (CHEERS) (see Supplementary Information S1) (1 USD = 6.46 CNY, 2021) (Husereau et al., 2013).

### Model Method

We extracted survival data from the ORIENT-15 trial for model building. The GetData Graph Digitizer (version 2.26; http://getdata-graph-digitizer.com/download.php) was used to obtain the Kaplan–Meier (KM) curve based on the PFS and OS of sintilimab combined with chemotherapy *versus* chemotherapy alone. We also referred to the algorithm of Guyot et al. who used the pseudo-individual patient’s data reconstructed by R software (version 4.1.0; https://www.r-project.org/) (Guyot et al., 2012). This was combined with the Akaike information criterion (AIC), Bayesian information criterion (BIC), and the visual method to select the optimal distribution from gamma, Weibull, exponential, log-normal, and log-logistic distributions after the reconstruction (Liu et al., 2019). Log-logistic and log-normal distribution can better simulate long-term survival for the survival curve (Supplementary Table S1). Details of model extrapolation are shown in Supplementary Figures S2,S3. Referring to the formula of Liu et al. (2021) to calculate the transition probability, we combined specific parameters of the model to estimate the dynamic transition probability between states for each cycle.

### Utility and Cost Estimates

During the follow-up period, the ORIENT-15 trial used the Quality of Life Questionnaire-Core 30 (QLQ-C30), the Quality of Life Questionnaire-Esophageal Cancer Module 18 (QLQ-OES18), and the Five Level EuroQol Five-Dimensional Questionnaire (EQ-5D-5L) to evaluate the patients’ quality of health. Since no specific questionnaire data were previously published, we referred to past studies to obtain the average health utility in terms of PFS and PD (PFS = 0.741 and PD = 0.581) of patients with esophageal cancer (Zhang et al., 2020). In order to simplify the model, we only considered Grade 3–4 adverse events (AEs) as the top three incidence rates according to ORIENT-15. We considered the loss of health utility caused by the occurrence of these adverse events (Haddad et al., 2020).

Only the direct costs of the medical expenses were considered. This included the cost of the drugs, subsequent treatment costs, management costs, follow-up costs, laboratory examination costs, and the major Grade 3/4 AEs. The drug prices were adjusted according to the local drug pricing and medical insurance policies after consulting with drug suppliers. The calculated drug costs were based on actual clinical trials. Once every three weeks, patients received immunotherapy (sintilimab, 3 mg/kg for patients weighing < 60 kg or 200 mg for patients weighing ≥ 60 kg *via* intravenous injection) with or without chemotherapy (5-fluorouracil, 175 mg/m^2^ and cisplatin, 75 mg/m^2^). We assumed that the average weight of a patient was 55 kg and the average body surface area was 1.68 m^2^ (Ward et al., 2017). The cost of AEs and other expenses came from the previously published literature (Murray et al., 2000; Nafees et al., 2008; Wu et al., 2012; Ding et al., 2020; Haddad et al., 2020; Pongchaiyakul et al., 2020; Yang et al., 2021). The estimated cost of each drug during the established time period is listed in Table 1. When the disease progressed, we assumed that all patients had a follow-up treatment. In this study, the additional treatment included camrelizumab (anti-PD-L1 agent), anlotinib (targeted drug therapy), and docetaxel (chemotherapy) (camrelizumab: 200 mg, intravenous injection; anlotinib: 12 mg orally daily for 14 days; docetaxel: 75 mg/m^2^, intravenous injection). All the drugs were given once every three weeks based on the National Comprehensive Cancer Network (NCCN) Guidelines version 1.2021 (NCCN, 2021).

**TABLE 1:** Model parameters: baseline values, ranges, and distributions for sensitivity analysis.

Variable	Baseline value	Range			Distribution	Reference
		Minimum	Maximum			
Log-logistic OS survival model in sintilimab + chemotherapy group	Shape = 1.9216, scale = 17.2448	ND	ND	—	ND	Model fitting
Log-logistic OS survival model in the chemotherapy group	Shape = 1.95315, scale = 12.30254	ND	ND	—	ND	Model fitting
Log-logistic PFS survival model in the sintilimab + chemotherapy group	Shape = 2.16, scale = 5.67	ND	ND	—	ND	Model fitting
Log-lnorm PFS survival model in the chemotherapy group	Meanlog = 2.19, sdlog = 0.903	ND	ND	—	ND	Model fitting
Risk for main adverse events						
Sintilimab + chemotherapy						
Leukopenia	0.17	0.136	0.204	0.034	Beta	ORIENT-15
Anemia	0.13	0.104	0.156	0.026	Beta	ORIENT-15
Neutropenia	0.3	0.24	0.36	0.06	Beta	ORIENT-15
Chemotherapy						
Leukopenia	0.07	0.056	0.084	0.014	Beta	ORIENT-15
Anemia	0	0	0	0	Beta	ORIENT-15
Neutropenia	0.12	0.096	0.144	0.024	Beta	ORIENT-15
Health utility scores						
Utility of PFS	0.741	0.593	0.889	0.1482	Beta	Lagergren et al. (2017)
Utility of PD	0.581	0.465	0.697	0.1162	Beta	Lagergren et al. (2017)
Cost, $/per cycle						
Sintilimab	301.78	241.424	362.136	60.356	Gamma	Local quotes
Cisplatin	15.644	12.5152	18.7728	3.1288	Gamma	Local quotes
Paclitaxel	103.814	83.0512	124.5768	20.7628	Gamma	Local quotes
5-Fluorouracil	45.64	36.512	54.768	9.128	Gamma	Local quotes
Laboratory test	157.5	126	189	31.5	Gamma	Rustgi and El-Serag, (2014)
Follow-up	59.2	47.36	71.04	11.84	Gamma	Uhlenhopp et al. (2020)
Administration	69.81	55.848	83.772	13.962	Gamma	Uhlenhopp et al. (2020)
Best supportive care	117.1	32.3	322.6	23.42	Gamma	Abnet et al. (2018)
Camrelizumab	463.4377968	370.7502	556.125356	92.6875594	Gamma	Local quotes
Anlotinib	639.556	511.6448	767.4672	127.9112	Gamma	Local quotes
Docetaxel	100	80	120	20	Gamma	Local quotes
Expenditures on main AEs, $						
Leukopenia	466	373	559	93.2	Gamma	Ilson and van Hillegersberg, (2018)
Anemia	531	425	638	106.2	Gamma	Ilson and van Hillegersberg, (2018)
Neutropenia	354	283	425	70.8	Gamma	Ilson and van Hillegersberg, (2018)
Disutility due to AEs						
Leukopenia	-0.0897	−0.07176	−0.10764	−0.01794	Beta	Napier et al. (2014)
Anemia	-0.073	−0.0876	−0.0584	−0.0146	Beta	Napier et al. (2014)
Neutropenia	-0.0897	−0.07176	−0.10764	−0.01794	Beta	Napier et al. (2014)
Risk for subsequent therapy						
Sintilimab + chemotherapy						
Anti-PD-(L)1 agent	0.34	0.272	0.408	—	Beta	ORIENT-15
Targeted drug therapy	0.13	0.104	0.156	—	Beta	ORIENT-15
Chemotherapy	0.12	0.096	0.144	—	Beta	ORIENT-15
Chemotherapy						
Anti-PD-(L)1 agent	0.29	0.232	0.348	—	Beta	ORIENT-15
Targeted drug therapy	0.055	0.044	0.066	—	Beta	ORIENT-15
Chemotherapy	0.055	0.044	0.066	—	Beta	ORIENT-15

Abbreviations: PFS, progression-free survival; PD, progressive disease; SAE, severe adverse event.

### Sensitivity Analysis

A one-way sensitivity analysis was performed to explore the influence of uncertain parameters on the ICER. The baseline value and 95 % confidence interval (CI) of the parameters were entered into the model. For parameters that could not obtain true uncertainty, we assumed that the change of the baseline value was ± 25 % to explore the impact on decision-making (Pei et al., 2021). Probability sensitivity analysis (PSA) was used to randomly sample all the parameters from a specified distribution to further explore the uncertainty and relevance of the model’s parameters. According to the parameter type, we selected the appropriate distribution for each uncertain parameter. Gamma distribution was selected to estimate the cost of the adverse reactions to drugs and treatment. Beta distribution was selected to estimate the health utility scores, including PFS, OS, and AEs. We performed a second-order Monte Carlo simulation of 10,000 iterations and generated a cost-benefit acceptability curve (CEAC) to demonstrate that sintilimab combined with chemotherapy was cost-effective with different WTP thresholds.

Based on the data from patients with combined positive scores of ≥ 10 for the expression of PD-L1 published in the ORIENT-15 clinical trial, we further conducted a cost-effective analysis of the PD-L1 combined positive score of 10 or more (CPS ≥ 10) group.

## Results

### Base-Case Analysis

From the perspective of the Chinese healthcare payers, the incremental cost of sintilimab plus chemotherapy for all patients was $21024.05, and in the CPS ≥ 10 group, it was $20974.23. The incremental health output was 1.06 LYs and 0.64 QALYs for all patients treated with sintilimab plus chemotherapy. The incremental health output was 1.10 LYs and 0.67 QALYs for the PD-1 CPS ≥ 10 group. The ICER per QALY for sintilimab plus chemotherapy *versus* chemotherapy alone was $21024.05 for all patients (Table 2).

**TABLE 2:** Base-case analysis results.

Strategy	Cost	Incr cost	LYs	Incr LYs	ICER/LYs	QALYs	Incr QALYs	ICER/QALYs
All patient group								
Chemotherapy	4190.37	—	0.73	—	—	0.53	—	—
Sintilimab + chemotherapy	17671.77	13481.39	1.79	1.06	12718.29	1.17	0.64	21024.05
PD-1 CPS≥10 group								
Chemotherapy	4300.36	—	0.75	—	—	0.55	—	—
Sintilimab + chemotherapy	18272.87	13972.51	1.85	1.10	12702.28	1.21	0.67	20974.23

Abbreviation: Incr cost, incremental cost; Lys, life-years; Incr Lys, incremental life-years; QALYs, quality-adjusted life-years; Incr QALYs, incremental quality-adjusted life-years; ICER, incremental cost-effectiveness ratio; PD-L1, programmed cell death-ligand 1

### Sensitivity Analysis

**FIGURE 1:**
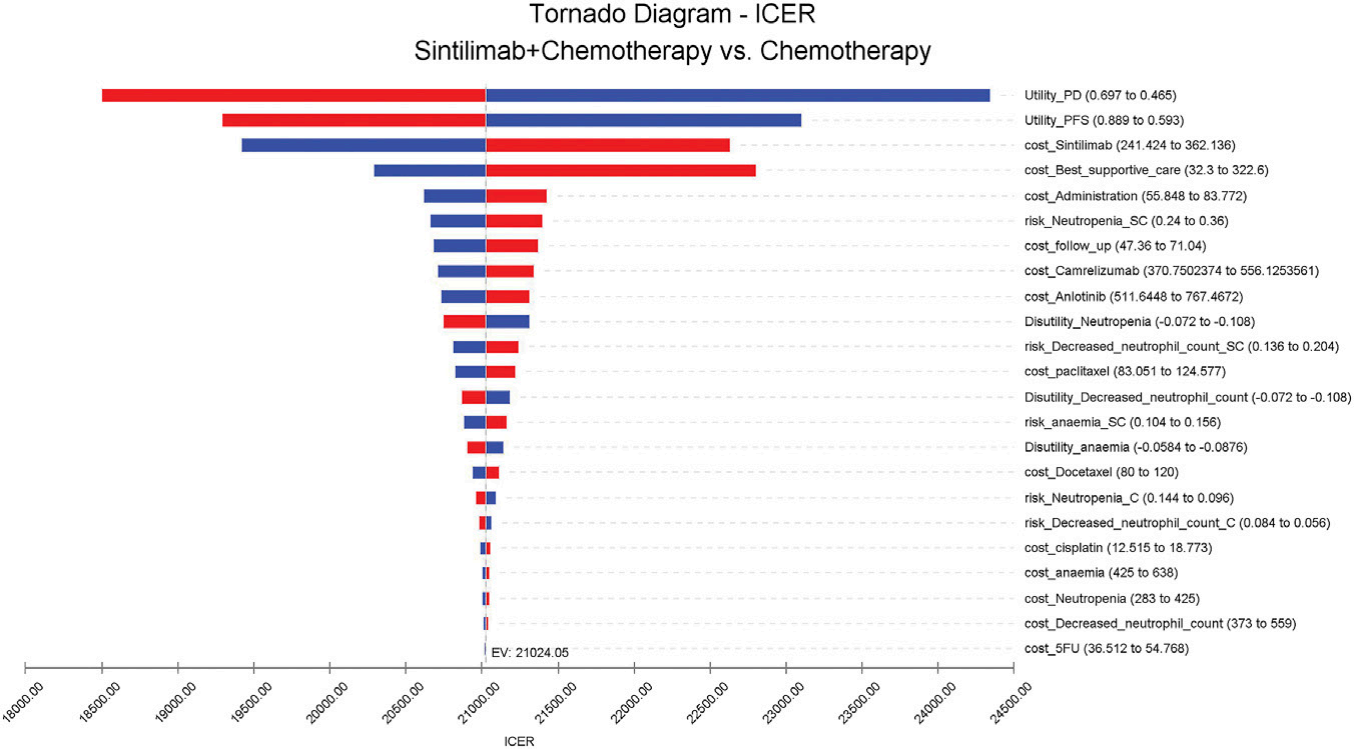
Tornado diagram for one-way sensitivity analysis in any PD-L1 expression group.

A one-way sensitivity analysis was used to test the robustness of the model. The influence of each parameter on the results was discussed within the variation range of input model parameters. The results are presented in the tornado diagram (Figure 1). The sensitivity analysis results demonstrated that the cost of sintilimab, the utility of PD, and the utility of PFS were the three primary factors with the greatest impact on the results for all the patients. Under the condition of a payment threshold of $37653 per QALY, when parameters varied within a given range, the ICER was still lower than the WTP of Chinese payers.

**FIGURE 2:**
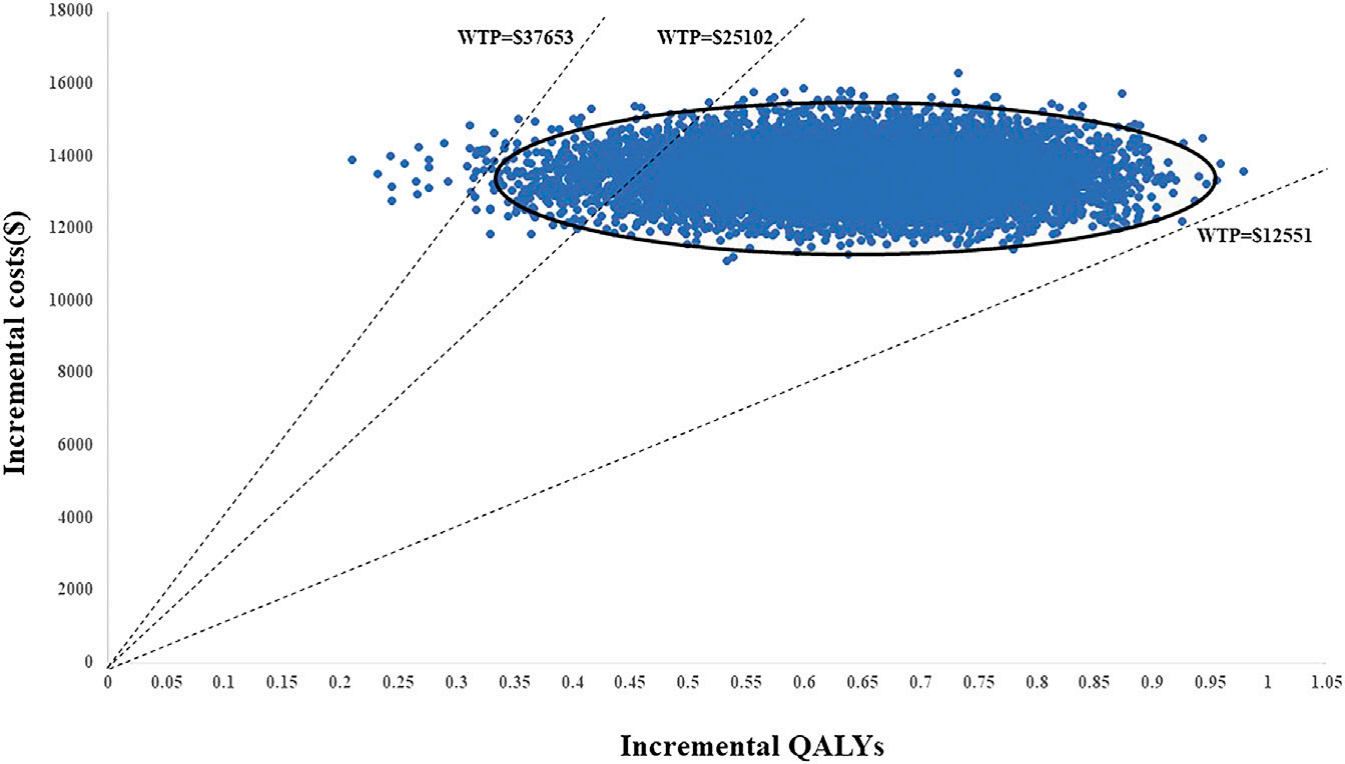
Incremental cost-effectiveness scatter plot (sintilimab + chemotherapy vs. chemotherapy).

**FIGURE 3:**
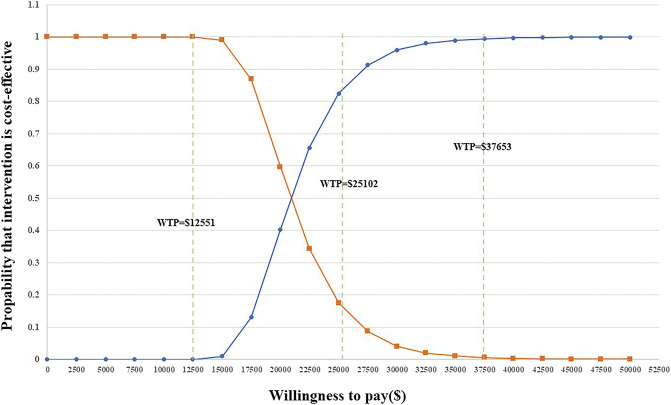
Acceptability curves for the choice of sintilimab + chemotherapy versus chemotherapy at different WTP thresholds in any PD-L1 expression group.

PSA was applied to test the bias of the multiple model parameters on the analysis results when the multiple model parameters changed simultaneously. The incremental cost-benefit scatter chart (Figure 3) displayed the results of Monte Carlo simulation. The cost-effectiveness acceptability curves (Figure 2) showed that the randomized patient group is compared with chemotherapy under the condition of a payment threshold of $37653 per QALY. For the combination therapy, the probability of sintilimab plus chemotherapy being cost-effective was 99.36 % .

### Scenario Analysis

In ORIENT-15, the use of sintilimab varied according to patient weight. Therefore, in this study, we assessed patients weighing ≥ 60 kg (42%) and < 60 kg (58%), and how this impacted sintilimab use. The data were obtained from the ORIENT-15 clinical trial. The sensitivity analysis demonstrated that the price of sintilimab had a greater impact on the results and that patient’s weight was an influencing factor. Therefore, we performed a cost-effectiveness analysis for patients with the assumed weight of 50 kg (3 mg/kg) and 60 kg (200 mg) with the ICER per QALY being $21933.11 and $19648.51, respectively.

In addition, we set the WTP to three times China’s GDP per capita in 2021. However, we wanted to explore whether the scenario can be cost-effective under different WTP thresholds. Therefore, we additionally assumed a WTP of twice ($25102 per QALY) China’s GDP per capita in 2021 ($12551). The probability of sintilimab plus chemotherapy being cost-effective was 83.12 % (WTP = $25102 per QALY) and 0.00 % (WTP = $37,653 per QALY) (Figure 3).

## Discussion

Sintilimab is an IgG4 monoclonal antibody that specifically binds to the PD-1 molecule on the surface of T cells, thereby inhibiting the PD-1/PD-L1 pathway, which prevents tumor immune tolerance and reactivates the anti-tumor activity of lymphocytes for the purpose of tumor treatment. The Chinese Society of Clinical Oncology (CSCO) Guidelines Conference 2022 included sintilimab in combination with chemotherapy as a potential first-line therapeutic approach for advanced gastric cancer and advanced esophageal squamous cancer.

In ORIENT-15, the clinical benefits of sintilimab combined with chemotherapy were demonstrated in patients with advanced esophageal cancer. This was regarded as a breakthrough treatment for esophageal cancer (Lu et al., 2022). However, the high cost of immunotherapy remains out of reach for most middle-class families. Finding a balance between price and effectiveness remains a key challenge. Clinicians may be discouraged from using immunotherapy with patients since it is often restricted to certain affluent groups. The income level of a patient was often considered when deciding whether to use immunotherapy (Elkin and Bach, 2010). An economic evaluation of immunotherapy, including sintilimab, could help to avoid squandering healthcare resources. In addition, it will guide physicians in selecting the best treatment options for this specific patient population.

Previous studies have analyzed the economic benefits of sintilimab combined with chemotherapy compared with chemotherapy alone as the first-line treatment of unresectable hepatocellular carcinoma, locally advanced or metastatic non-squamous (Peng et al., 2022; Rui et al., 2022). The economic evaluation of sintilimab as the primary treatment for advanced esophageal cancer was lacking. Therefore, a detailed evaluation of its costs and health outcomes was required which we aimed to provide.

From the perspective of the Chinese healthcare payers, based on the clinical trial results of ORIENT-15, we established a multi-state Markov model to evaluate the economic differences of sintilimab plus chemotherapy and platinum bimodal drug therapy. In ORIENT-15, KM survival curves for all patients (any PD-L1 expression) and PD-L1 expression positive (CPS≥10) were not significantly different in OS and PFS. In our study, for locally advanced or metastatic esophageal squamous cell carcinoma, the combination of sintilimab and chemotherapy was cost-effective in the total population and in PD-L1-positive population at a WTP threshold of $37,653. Sensitivity analysis suggested that the price of sintilimab had a large effect on the results. Therefore, we conducted a scenario analysis of the factors that may influence the price of sintilimab, such as weight, and the results suggested that it would be cost-effective regardless of weight. The cost-effectiveness analysis has different results under different WTP criteria, but it is encouraging to note that even at two to three times China’s GDP in 2021, there was still at least an 83.12 % probability that sintilimab plus chemotherapy in locally advanced or metastatic esophageal squamous cell carcinoma was cost-effective.

We did not find other studies on the cost-effectiveness analysis related to sintilimab and esophageal cancer, but there were three cost-effectiveness analyses of PD-L1 inhibitors related to esophageal cancer.

Two studies suggest that pembrolizumab was not cost-effective in advanced esophageal cancer, with Zhan et al. (2022) suggesting an increased cost of $37,201.68 for pembrolizumab compared to chemotherapy alone while obtaining a QALY of 0.23. Zhu et al. (2022) suggested an ICER per QALY for pembrolizumab plus chemotherapy compared to chemotherapy of $550,211 in the United States and China were $244,580/QALY and $258,261/QALY, respectively. Pembrolizumab plus chemotherapy yielded 0.386–0.607 QALYs (0.781–1.195 LYs) compared with chemotherapy alone, and both studies had well above the standard WTP. These two studies had no cost effect due to the much higher price of pembrolizumab than the price of the chemotherapy group. In our study, sintilimab plus chemotherapy obtained QALY values of 0.64–0.67 compared to chemotherapy alone, and with the low price of sintilimab relative to pembrolizumab, sintilimab plus chemotherapy was therefore more cost-effective for first-line recommendations in locally advanced or metastatic esophageal squamous cell carcinoma. In addition, Cai et al. (2021) demonstrated that camrelizumab was cost-effective as a second-line regimen compared to chemotherapy in locally advanced or metastatic esophageal squamous cell carcinoma (incremental cost of $1,439.64; added 0.36 QALYs; ICER of $3,999 per QALY). According to his findings, the price of the drug was not significant. The main reason for its cost-effectiveness was the low price of camrelizumab ($432 at a dose of 200 mg) due to its inclusion in China’s national health insurance reimbursement (http://www.nhsa.gov.cn/art/2020/12/28/art_14_4221.html) and the similarly low price of its control chemotherapy drugs (docetaxel: $1.77; irinotecan: $1.64). Unfortunately, the study was based on second-line treatment for locally advanced/metastatic esophageal squamous cell carcinoma, and the chemotherapy regimens used as controls were inconsistent. Therefore, we were unable to make a direct comparison between camrelizumab and sintilimab to determine which was more cost-effective. Large-scale future clinical trials with long follow-up periods are needed to facilitate a comparison of the advantages and disadvantages of the two immunotherapies.

### Limitations

This study had some limitations. First, ORIENT-15 was a phase III randomized controlled trial, and we used this model to simplify the study. For instance, regarding the AEs, we selected the topmost three to four main AEs that grade 3 or higher. Second, the data originated from the ORIENT-15 trial. Due to the limitation of the number of patients included in the trial, we could not perform a larger-scale analysis and the trial did not provide follow-up survival data for patients. We relied on the survival data from the trial and performed a reasonable extrapolation to predict the long-term survival of patients. This will inevitably vary from the data of real-world patients obtained through regular follow-ups. Third, since ORIENT-15 does not disclose the specific health data of patients, our PFS and PD utility were derived from previously published related studies. This may be different from the real-life situation. Fourth, we only considered the cost impact and utility reduction caused by the three main AEs. The utility reduction caused by specific AEs was derived from other published literature works. Fifth, the treatment plan of the trial, and especially the follow-up treatment of patients, will be adjusted appropriately according to the specific situation. For instance, we did not find specific information about follow-up treatment in the ORIENT-15 data, so we assumed several follow-up treatment options, which affected the treatment impact of the two groups to a certain extent. The results of the study were inadequate due to several factors, and more accurate data could be obtained in the future by increasing the sample size and with a longer follow-up period. Therefore, more clinical trials are required in the future to reduce the study population, follow-up treatment, and other factors that impact the results.

## Conclusion

Overall, from the perspective of the Chinese health-care payers, sintilimab plus chemotherapy should be considered as the first-line treatment for patients with locally advanced or metastatic esophageal squamous cell carcinoma. Compared with chemotherapy, the combination therapy would be a more cost-effective choice.

## Data Availability

The original contributions presented in the study are included in the article/Supplementary Material; further inquiries can be directed to the corresponding author.
