# Supplementary material for: Cost-Effectiveness Analysis of Sintilimab Combined with Chemotherapy Versus Chemotherapy Alone as the First-Line Treatment for Advanced Esophageal Cancer
Source: Front Pharmacol. 2022 Nov 28;13:934275. doi: 10.3389/fphar.2022.934275 (PMC9742528; doi:10.3389/fphar.2022.934275)
Supplement: Supplementary file 1 [file DataSheet1.docx]

eFigure1: Markov state transition probability diagram.


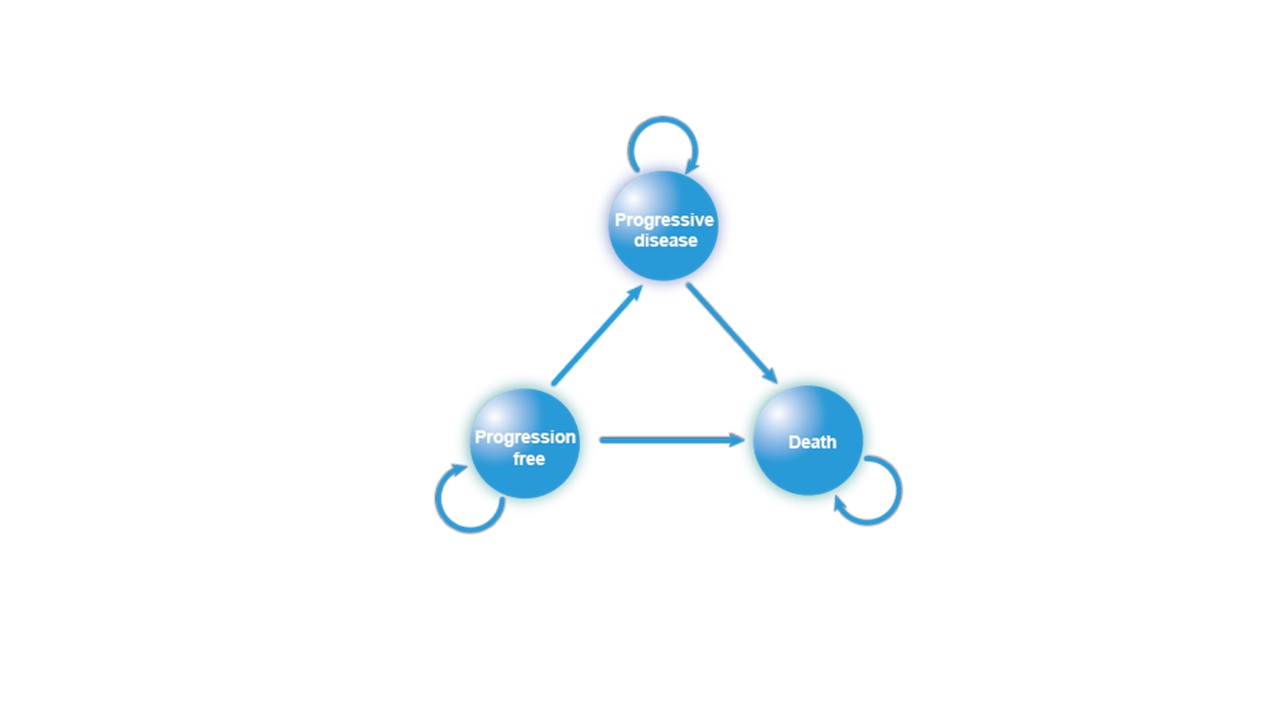


eFigure2. Fitting and extrapolation of Kaplan Meier survival curve for PFS


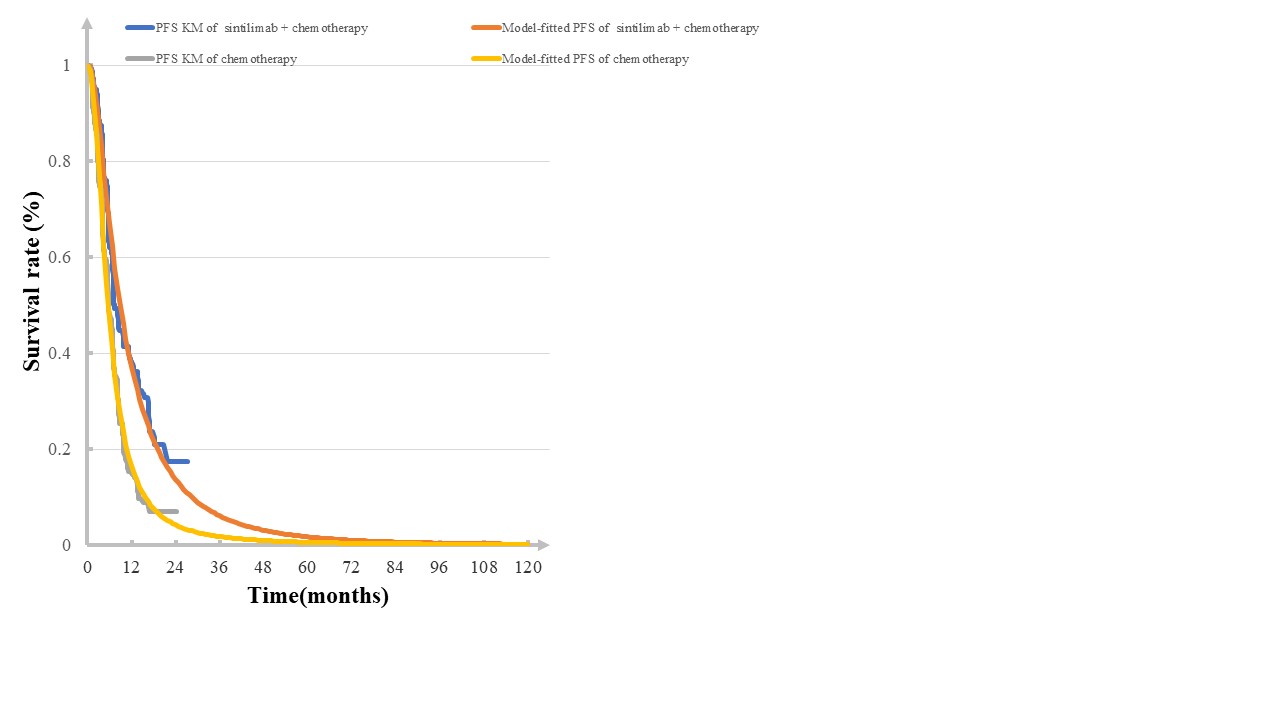


eFigure3. Fitting and extrapolation of Kaplan Meier survival curve for OS


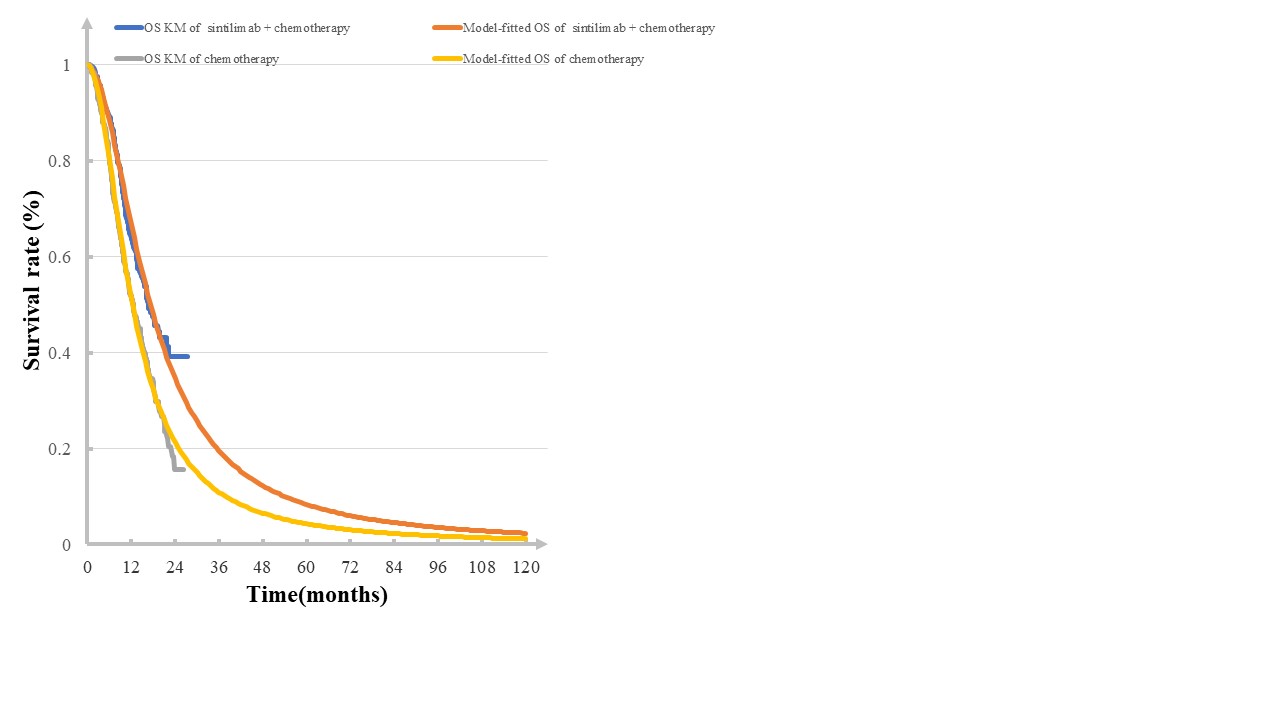


eTable1.AIC and BIC scores of fitted distribution in CPS≥10 group and any PD-L1 expression group

eTable1.AIC and BIC scores of fitted distribution in all patients from ORIENT-15

| Distribution | OS of sintilimab + chemotherapy | | | | | OS of chemotherapy | | | | PFS of sintilimab + chemotherapy | | | | PFS of chemotherapy | | | |
| --- | --- | --- | --- | --- | --- | --- | --- | --- | --- | --- | --- | --- | --- | --- | --- | --- | --- |
| **All patients** | AIC | | BIC | | AIC | | | BIC | | AIC | | BIC | | AIC | | BIC | |
| Exponential | 1265.047 | | 1272.627 | | 1562.295 | | | 1569.906 | | 1385.722 | | 1393.302 | | 1510.342 | | 1517.952 | |
| Gamma | 1229.011 | | 1240.381 | | 1519.604 | | | 1531.319 | | 1350.768 | | 1362.137 | | 1448.706 | | 1460.121 | |
| Weibull | 1233.265 | | 1244.635 | | 1520.855 | | | 1532.27 | | 1360.32 | | 1371.69 | | 1459.215 | | 1470.631 | |
| Log-normal | 1225.849 | | 1237.218 | | 1521.204 | | | 1532.619 | | 1333.479 | | 1344.849 | | 1445.126 | | 1456.541 | |
| Log-logistic | 1225.598 | | 1236.968 | | 1517.771 | | | 1529.186 | | 1335.401 | | 1346.771 | | 1442.739 | | 1454.155 | |
| **PD-1 CPS≥10 group** | | AIC | | BIC | | | AIC | | BIC | | AIC | | BIC | | AIC | | BIC |
| Exponential | | 715.0197 | | 721.4925 | | | 894.7654 | | 901.2908 | | 779.3615 | | 785.8344 | | 825.5596 | | 832.085 |
| Gamma | | 688.1983 | | 697.9076 | | | 867.4808 | | 877.2689 | | 758.4552 | | 768.1645 | | 787.5084 | | 797.2965 |
| Weibull | | 690.7679 | | 700.4772 | | | 870.217 | | 880.0051 | | 764.3265 | | 774.0358 | | 793.8774 | | 803.6655 |
| Log-normal | | 686.5863 | | 696.2956 | | | 865.6089 | | 875.3969 | | 746.6873 | | 756.3966 | | 783.8878 | | 793.6759 |
| Log-logistic | | 686.8066 | | 696.5159 | | | 867.293 | | 877.0811 | | 750.3732 | | 760.0826 | | 783.0432 | | 792.8313 |

The results showed that the Log-logistic and Log-normal distribution had the lowest AIC and BIC values, so we used Log-logistic and Log-normal to fit the OS and PFS curves in our analysis.

Abbreviation: OS=Overall survival; PFS= Progression-free survival; AIC= Akaike information criterion; BIC= Bayesian information criterion. PD-L1;Programmed cell death-Ligand 1.
